# Intergroup Variation of Social Relationships in Wild Vervet Monkeys: A Dynamic Network Approach

**DOI:** 10.3389/fpsyg.2016.00915

**Published:** 2016-06-21

**Authors:** Christèle Borgeaud, Sebastian Sosa, Redouan Bshary, Cédric Sueur, Erica van de Waal

**Affiliations:** ^1^Laboratory of Eco-Ethology, Institute of Biology, University of NeuchâtelNeuchâtel, Switzerland; ^2^Inkawu Vervet Project, Mawana Game ReserveKwaZulu Natal, South Africa; ^3^Adaptive Behavior and Interaction Research Group, University of BarcelonaBarcelona, Spain; ^4^Département Ecologie, Physiologie et Ethologie, Centre National de la Recherche ScientifiqueStrasbourg, France; ^5^Institut Pluridisciplinaire Hubert Curien, Université de StrasbourgStrasbourg, France; ^6^Anthropological Institute and Museum, University of ZurichZurich, Switzerland

**Keywords:** social network, dynamics of relationships, RSiena, group composition variation, vervet monkeys

## Abstract

Social network analysis is a powerful tool that enables us to describe and quantify relationships between individuals. So far most of the studies rely on the analyses of various network snapshots, but do not capture changes over time. Here we use a stochastic actor-oriented model (SAOM) to test both the structure and the dynamics of relationships of three groups of wild vervet monkeys. We found that triadic closure (i.e., the friend of a friend is a friend) was significant in all three groups while degree popularity (i.e., the willingness to associate with individuals with high degree of connections) was significant in only two groups (AK, BD). The structure and dynamics of relationships according to the attributes of sex, matrilineand age differed significantly among groups. With respect to the structure, when analyzing the likelihood of bonds according to the different attributes, we found that individuals associate themselves preferably to individuals of the same sex only in two groups (AK, NH), while significant results for attachment to individuals of the same matriline were found also in two groups (BD, NH). With respect to the dynamics, i.e., how quickly relationships are modified, we found in two groups (AK, BD) that females' relationships were more prone to variation than males.' In the BD group, relationships within high-ranking matrilines were less stable than low-ranking ones while in the NH group, juveniles' relationships were also less stable than adults' ones. The intergroup variation indicates that establishing species-specific or even population specific characteristics of social networks for later between-species comparisons will be challenging. Although, such variation could also indicate some methodological issue, we are quite confident that data was collected similarly within the different groups. Our study therefore provides a potential new method to quantify social complexity according to natural demographic variation.

## Introduction

Social network analysis is a method that is used to describe and quantify relationship patterns within a group. Such metrics can be applied at an individual, group or species level. During the last decade, social network analysis has become increasingly popular, especially in primatology (Silk et al., 2003, 2010; Flack et al., 2006; Sueur and Petit, 2008; Henzi et al., 2009). However, most previous studies considered a network to be a static structure that does not vary over time. The few studies that integrated temporal variation focused on dyadic relationships or at the group level and compared networks at different periods (Silk et al., 2006a; Henzi et al., 2009). Such a dynamic approach is necessary if we aim at quantifying network instability and hence the need of an individual to monitor and update its knowledge about its own and also third party relationships. One study tested the influence of natural “knock-outs” within the group (Barrett et al., 2012) and measured their effects in term of entropy (i.e., uncertainty reduction). Another one used 20 years of data on a clan of spotted hyenas to understand the effect of rainfall and abundance of prey on the network structure (Ilany et al., 2015). In a parallel publication (Borgeaud et al., in preparation) on wild vervet monkeys, we also made a first step forward toward the analysis of a network dynamics by considering the influence of demographic variation (i.e., the number of individuals entering and leaving the group) on the individual centrality and on the dyadic relationship stability. Results suggested that, despite some intergroup variation, demographic variation of females, and juveniles have a stronger influence than males on both centrality and the relationship stability. This seems logical knowing that, in vervet monkeys, females remain generally in their natal group for their entire life and form strong and long-lasting bonds with their kin, while males migrate throughout their lives (Cheney and Seyfarth, 1990). However, despite the development of new analytical methods, studies that took into consideration changes over time within a network remain scarce (see Pinter-Wollman et al., 2013 for a review).

Explaining cooperative behaviors that benefit the recipient at some cost to the donor (i.e., helping based on investments) has been a great challenge. Both the kin selection (Hamilton, 1964) and the reciprocity (Trivers, 1971) concepts provided an evolutionary explanation to helping, respectively within related and unrelated individuals. Social network analyses have been proposed as a powerful tool to describe how individuals influence each other within a network and how these relationships evolve over time. Ultimately understanding the dynamics of these relationships could help explain how cooperation evolves. For example, triadic closure (i.e., the hypothesis that an individual is more likely to create bonds with the friends of its friends) may facilitate the formation of cohesive sub-/groups and consequently cooperation within a social group (Granovetter, 1973; Lusseau et al., 2006; Easley and Kleinberg, 2010). The process that describes how individuals associate preferably to individuals with high centrality is called degree popularity (Barabási and Albert, 1999) and some studies found that high-ranking individuals are usually more central within a grooming and proximity network (see Schino, 2001 for a meta-analysis; Kanngiesser et al., 2011; Sueur et al., 2011a; Borgeaud et al., in preparation). This supports Seyfarth's theory (1977), which suggests that grooming could be exchanged against coalitionary support and that individuals should compete to associate with high-ranking individuals as they provided better support during conflicts or as tolerance in the vicinity of food resources increase with grooming exchanged. In this way, individuals attracted to central individuals might have a better fitness than other less strategic individuals. Another interesting measurement is the assortativity of relationships based on individual traits which is called homophily (see McPherson et al., 2001 for a review). Examples include space use in sea lions (Wolf et al., 2007), sex and age-related relationships in dolphins (Lusseau and Newman, 2004), and personality in sticklebacks (Pike et al., 2008). Homophily might also increase an individual's fitness. For example, playing behavior between juveniles decreases the risk of injuries (Shimada and Sueur, 2014) and personality or sex segregation increases food research efficiency (Ruckstuhl and Kokko, 2002; Dyer et al., 2009). In primates, some studies report that, except for kin who usually forms the strongest bonds (Chapais, 2001; Silk et al., 2006a,b, 2010, 2012), unrelated individuals of similar rank or age also form long-lasting relationships (Silk et al., 2006a, 2010, 2012). Such bondedness could be explained through familiarity and eventually paternal kinship (Seyfarth and Cheney, 2012) but also personality (Massen and Koski, 2014). It has been reported that the quality of such bonds have an influence of an individual's fitness such as its longevity and offspring survival (Silk et al., 2003, 2009, 2010) resulting in the selection of such social strategies but a lot of studies analyzed such relationships as being part of a static network. Hence it would be important to apply a more dynamic approach to the analyses of relationships quality which evolve naturally over time (Ilany et al., 2015).

One method that has been developed is the Siena model (for Simulation Investigation for Empirical Network Analysis, Snijders, 2001; Blonder et al., 2012; Pinter-Wollman et al., 2013; Ilany et al., 2015; Pasquaretta et al., 2016), available in the R package RSiena. This stochastic actor-based model aims to give a realistic representation of the dependence between the formation and also termination of different network ties. It therefore allowed us to examine how network processes and covariates influence the probability of individuals changing their network ties according to their attributes over time (Burk et al., 2007; Snijders et al., 2010). By applying these analyses on three wild groups of vervet monkeys over a period of 2 years, we aimed at describing the dynamics of their social network (in terms of grooming and proximities relationships) according to the natural demographic variation. Vervet monkeys represent an ideal model as, in addition to natural disappearance, every year a new generation of infants gets integrated. Native sub-adult males leave the group once they have reached sexual maturity and adult males migrate throughout their whole life joining and leaving multiple groups (Cheney and Seyfarth, 1990).

**Figure 1:**
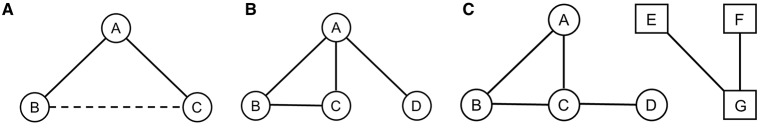
**Representations of (A) Triadic closure: If A and B are connected, the probability of B and C being connected is increased; (B) Degree popularity: A being more connected has a higher degree popularity than B, C, and D; (C) Homophily: A, B, C, and D are more connected to each other as they have similar attribute characteristics such as hierarchy for example than they are connected to E, F, and G who themselves have similar attribute characteristics**.

RSiena is a powerful program allowing us to answer many questions about the mutually dependent dynamics of networks and attributes (behavior, individual characteristics, etc.) of the individual actors in the network. The RSiena approach allows testing of a great variety of potentially interesting network characteristics such as triadic closure, homophily, and rate effect, which analyses the relationships' stability according to various individual attributes. This approach allowed us to assess how the relationships' quality (i.e., based on grooming and proximity data) evolves in function of the natural demographic variations. First, we tested the effect of triadic closure (Figure 1A) and degree popularity as well as the temporal persistence of these effects. As vervet monkeys are a highly social species that shows some level of cooperation (Cheney and Seyfarth, 1990; Borgeaud and Bshary, 2015), we expected triadic closure to be present in all three groups. Specifically, the triadic closure effect will assess whether new incoming individuals developing relationships with specific individuals will also develop relationships with their “friends.” Triadic closure is a good model to understand how networks will evolve over time. While simple graph theory tends to analyze networks at one point in time, applying the triadic closure principle can predict the development of ties within a network and shows the progression of connectivity (Easley and Kleinberg, 2010). We also tested the effect of degree popularity (Figure 1B): as high-ranking individuals offer better support in case of conflict (Cheney and Seyfarth, 1990) and could also confer some protection when spending time in their proximity (Watts, 2002; Cheney and Seyfarth, 2007) they should be preferred targets for bonding attempts and hence should receive disproportionate amounts of grooming. Therefore, new incomers would challenge existing links between group members and in this case it might result in detectable variation of central/high ranking individuals' position within the network (Cheney and Seyfarth, 1990; Borgeaud et al., in preparation). We also tested homophilic bonds (Figure 1C) to know if individuals preferably associate with individuals of similar attributes such as sex, matriline, hierarchy, and age. As females are the philopatric sex and normally remain in their natal group throughout their lives (Cheney and Seyfarth, 1990), we expected them to form stronger bonds with other females rather than with males. As juveniles from the same generation spend at least 4 years within the same group before a potential migration (i.e., for the males) and as adult females have spent many years within the same group (Cheney and Seyfarth, 1990), we expected individuals from similar age to form stronger bonds (Silk et al., 2010). We also expected individuals of similar rank in the hierarchy to form stronger bonds than individuals of distant rank as usually neighboring ranks are more closely related (Cheney and Seyfarth, 1990). Furthermore, as hypothesized by Seyfarth (1977), if high-ranking females are indeed preferred grooming partners, competition may limit the access to high-ranking partners only to neighboring rank individuals (Silk et al., 2006a,b). Finally, we examined how the different group members' relationships according to the same individual attributes are prone to variation over time. As indicated by Silk et al. (2010), adult female baboons form strong and stable bonds with their kin and with females of similar age. We therefore expected the same for female vervet monkeys while males' relationships should be more prone to variation.

## Methods

### Study groups

The study was conducted from January 2012 until December 2013 at the Inkawu Vervet Project, Mawana game reserve (S 28° 00.327; E 031° 12.348), Kwazulu Natal, South Africa. Subjects were three habituated groups of wild vervet monkeys. All individuals were recognized individually through facial and body features. Observers were all requested to pass an identification test and data were collected only if the identity of the individual was certain. We considered females as adults as soon as they had their first infant and males as adults once they migrated from their natal group. Individuals were considered as juveniles (including sub-adults, i.e., generally 3 years old females before they give birth and males before they emigrate) from the age of 1 until adulthood and as infants up to 1 year old. All three groups had been regularly followed since 2010, allowing us to have a good estimation of their age, although for the analyses we considered only two age categories: adult or juvenile while infants were excluded. The size of the Ankhase (AK) group excluding infants varied from 26 to 33 individuals (including from 4 to 7 adult males, 6 to 8 adult females, and 12 to 19 juveniles), Baie Dankie (BD) group varied from 36 to 48 individuals (4 to 5 adult males, 11 to 14 adult females, and 19 to 33 juveniles), and the Noha (NH) group varied from 25 to 41 individuals (2 to 7 adult males, 11 to 12 adult females, and 11 to 25 juveniles) (see Table 1 for group composition). Hierarchy was assessed by the creation of matrices based on dyadic aggressive interactions (i.e., winner-loser) occurring either in a natural context or around various food experiments. Rank relationships were assessed through the “de Vries” (1998) methodology. The “I&SI” method of de Vries (1998), in which parts of the hierarchy that are unresolved by the “I” method (Slater, 1961, which minimizes the number of inconsistencies) are decided by minimizing the sum of the rank differences between individuals whose ranks are inconsistent, gave us a list of individuals from the most to the less dominant one. The female hierarchy remained stable during the study period, while the male one was highly variable across 3 months periods.

**Table 1:** **Group composition**.

	**Group**		
	**AK**	**BD**	**NH**
Adult males	4–7	4–5	2–7
Adult females	6–8	11–14	11–12
Juveniles and subadults	12–19	19–33	11–25
Total	26–33	36–48	25–41

### Data collection

Grooming, 1 and 5 m proximity data were collected through the method of scan sampling (Altmann, 1974) during two full days per week per group. Every 30 min and during a window of 10 min, observers walked within the group to collect the behavior of a maximum number of individuals (except infants). For each scanned individual the identity of all the individuals present within 1 and 5 m of it was also recorded. As data were collected by multiple observers, an inter-observer reliability test was performed for each observer and for each category of data to reduce any bias. The threshold of reliability was set to a minimum of 80%. In total we analyzed 3 months periods over 2 years which equals a total of 8 different periods. In the AK group we collected 31,661 scans, in BD 28,548 and in NH 28,448. Data were collected on handheld computers (Palm Zire 22 or TX, PDA 32 and Pocket pc HP Travel Companion iPAQ rx5935) equipped with the Pendragon 5.1 software.

### SIENA model and statistical analyses

SIENA Model (Simulation Investigation for Empirical Network Analysis; Snijders et al., 2010) is a log-linear dynamic model based on Markov processes that allows longitudinal network analysis. It uses an iterative stochastic simulation algorithm in three phases (calculating, updating, and re-calculating) as a Diffusion model updating statistical values after each iteration, making it a powerful method to find significant effects (effects that are greater than expected based on random models) and decreasing probabilities to find false positives (an effect that does really not exist) or false negatives (absence of effect that should be present).

Concerning actors, the model is based on the purposive action. Actors are considered as aware of the state of the network as a whole. They make choices and they can opt for creating, maintaining, or deleting an association in order to optimize their position within the group. These choices are done independently but can be constrained by endogen effects (i.e., relational structuring processes that depend on relational choices made by all the actors but independent from individual characteristics), hexogen effects (i.e., individual attributes such as sex or age), and some random effects.

As for the network, SIENA proposes a statistical model for longitudinal data analysis that requires at least two observations of the state of a network at two consecutive moments. The model supposes that some observations are missing between the two moments and that changes occur on a linear time basis through small steps between the two states observed. Thus, the model is based on Markov chains with linear time in which the future state of the network is linked to the previous state.

Siena only runs on binary matrices (existence or absence of links). In order to turn our valued matrices into binary matrices we used the protocol established by Fedurek et al. (2013) to create a mutual preferred social patterns index based on multiple social indices (i.e., grooming and proximity). The first step of this protocol consists of establishing a threshold for each one of the eight matrices for the three behaviors (grooming, 1 and 5 m proximity). The threshold is based on one-third standard deviation larger than the average for each behavioral matrix. The second and final step consists in considering the dyads as mutual preferred social partners if they were mutual associates for at least two of the three different behavioral matrices at a given time point (Fedurek et al., 2013; Levé et al., 2016). We repeated this protocol for each of the three groups.

The dependent variable here is the change in network relations with an analysis of factors influencing network changes over time. This network modeling aims to explain the network from the links and the actors it is composed of and also to explain the emergence, the pattern, and the evolution of relations within the network. To determine whether effects are significant or not, RSiena applies a stochastic simulation algorithm. The procedure consists of simulating many networks to observe if the value of the effects in these simulated networks is different or not from the observed network. Simulation allows us to obtain two parameters, the estimate and the standard error. To obtain the significance of the effect we performed a Wald-type test (based on the parameter estimate and the covariance matrix). Under the null hypothesis that parameter is zero with approximately a standard normal distribution. See Ripley et al. (2011) for more information about this procedure.

The network evaluation function (analysis of the probability of changes in the links according to some patterns called factors in RSiena) for an actor *I* is defined by:

(1) $$\begin{array}{l} f_{i}^{net}\left(x\right)\;=\;\underset{k}{\sum }\beta_{k}^{net}s_{ik}^{net}\left(x\right) \end{array}$$

Where $\beta_{k}^{net}$ are the parameters and $s_{ik}^{net}$ are the effects chosen by the user (in this research the “TransTrip,” “ InPop,” and “SimX” effects are described above respectively in Equations (2–4).

The analytical protocol consisted in adding the effects one by one, and testing the significance of the effect after each addition. The effect was retained when significant, otherwise it was simply removed from the model.

The first effect tested in the model was one potential structural effects: the “TransTrip,” which give information about phenomenon of triadic closure process (TC):

- TransTrip (i.e., TC) effect analyses individuals' transitivity (i.e.,). It is calculated by the number of transitive triplets among relations of *i* (*i* is linked to *j* and *h*, and these are linked to each other). It describes the ≪ friends of my friends are my friends ≫ phenomenon. The TransTrip effect formula is as follows:
  (2) $$\begin{array}{l} s_{ik}^{net}\left(x\right)=\underset{j,h}{\sum }x_{ij}x_{ih}x_{jh} \end{array}$$

For this effect the contribution of the relation *i* → *j* is proportional to the total number of transitive triplets formed, which can be (*i*→*j*→*h*; *i*→*h*) or (*i*→*h*→*j*; *i*→*j*).

The second effect tested in the model was another potential structural effects: “InPop,” which give information about growth-preferential association (PA).

- The inPop (i.e., PA) effect analyses individuals' ≪ popularity ≫ [i.e., defined by summing relations received by actors *j (degree)* whom *i* is linked to]. In our case as the networks are undirected we can consider this effect as degree popularity. It is calculated by the sum of in-degrees of the individual whom *i* is linked to. Popularity effect discloses individuals' preference to be linked to popular actors (i.e., individuals with highest degrees receive more incoming links). The inPop effect formula is as follows:
  (3) $$\begin{array}{l} s_{ik}^{net}\left(x\right)=\underset{i}{\sum }x_{ij}\underset{h}{\sum }x_{hj} \end{array}$$

Then we investigated the influence of covariate factors one by one by analyzing the “SimX” effects according to sex, matriline hierarchical rank and age, which give information about the tendency of individuals to create relations with individuals with similar attributes. This effect can be seen as an analysis of homophily or heterophily processes. Calculation details of this effect are described above and further information can be found in SIENA manual (Ripley et al., 2011).

- The covariate-related similarity (SimX) effect is the sum of centered similarity scores ${sim}_{ij}^{v}$ between *i* and the other actors *j* to whom he is tied according to the covariate *v*. The SimX effect formula is as follows:
  (4) $$\begin{array}{l} s_{ik}^{net}\left(x\right)=\underset{j}{\sum }x_{ij}\left(sim_{ij}^{v}-{\hat{sim}}^{v}\right) \end{array}$$

Where ${\hat{\text{sim}}}^{\text{v}}$ is the mean of all similarity scores.

For each one of this “SimX” effects we added at the same time the “Covariate-ego × alter” effect in order to control unequal ties between groups. The “Covariate-ego × alter” effect is simply the product of I's covariate and sum of his alters. To consider the effect as significant, both “SimX” and “Covariate-ego × alter” effects have to be significant.

Finally, we investigated the rate function effect according to sex, matriline, hierarchy and age one by one. The network rate function analyses how fast interactions change according to individual attributes (e.g., females have higher rate changes than males) for an actor *i.* This function is restricted to positive values as product of exponential elements. It can be defined by:

(5) $$\begin{array}{rcl} \lambda_{i}^{net}\left(\rho ,\alpha ,x,m\right)=\lambda_{i1}^{net}\lambda_{i2}^{net}\lambda_{i3}^{net},\;for\;x\; & = & \;x\left(t\right),\;t_{m}\;\leq \;t\\ < & \;t_{m+1} \end{array}$$

With $\lambda_{i1}^{net}\;=\;\rho_{m}^{net}$ representing the dependence of the period, $\lambda_{i2}^{net}\;=\text{ exp}\left(\underset{h}{\sum }\alpha_{h}v_{hi}\right)$ representing the effect of actor covariates (*v*_*hi*_ as the factor and α_*h*_ as the dependence of the degree) and $\lambda_{i3}^{net}\;$= exp(α_*h*_ + *x_i_*) representing the contribution of the degree (actor's personal network). Where ρ is the basic rate parameter, α is the dependence of the degree, *m* is the period (number of observation minus one), and *t* is the time point.

Models that included all the effects did not provide accurate goodness of fit analyses. For each group, we therefore realized a global model built up step by step by adding and testing the significance of one effect at a time. Once we obtained the final model for each group, we ran a goodness-of-fit test to assess if our model was significantly different from the observational data. We run a one-tailed Monte Carlo Mahalanobis distance test. After controlling for unequal ties between groups, such methodology led to the disappearance of the “hierarchy” attribute effect within the whole model and the “age” effect when testing the presence of homophilic bonds (Table 2). We therefore present only significant results below but discuss the absence of these effects within our global model further below. Goodness of fit plots for the degree distribution, the geodesic distribution and the triad census for each group are also presented in the Supplementary Figure 1.

**Table 2:** **Stochastic actor-oriented model, results summary**.

	**AK**						**BD**						**NH**					
	**Estimate**	**Std error**	***t* ratio**	**X2**	**DF**	***P*-value**	**Estimate**	**Std error**	***t* ratio**	**X2**	**DF**	***P*-value**	**Estimate**	**Std error**	***t* ratio**	**X2**	**DF**	***P* value**
Transitive triplets	0.08	0.04	0.11	4.794	1	**0.029**	0.24	0.05	0.08	21.573	1	**<0.001**	0.27	0.04	−0.02	53.561	1	**<0.001**
Degree popularity	−1.02	0.18	0.12	3.918	1	**0.048**	0.06	0.03	0.09	4.228	1	**0.039**				0.131	1	0.718
Same sex	−0.5	0.17	0.1	8.615	1	**0.003**				0.003	1	0.986	-1.84	0.4	-0.01	21.719	1	**<0.001**
Ego sex effect	1.06	0.28	0.01	14.514	1	**<0.001**				0.002	1	0.963	3.83	0.76	0.03	25.452	*1*	**<*0.001***
Same matriline				28.608	1	< 0.001	0.83	0.13	0.01	41.833	1	**<0.001**	0.98	0.12	0.03	71.463	1	**<0.001**
Ego matriline effect				1.235	1	0.266	0.01	< 0.001	0.05	7.045	1	**0.008**	0.02	0.01	< −0.001	8.309	1	**0.004**
Same hierarchy				2.715	1	0.099				5.486	1	0.019				19.866	1	< 0.001
Ego hierarchy effect				0.156	1	0.692				0.017	1	0.898				0.423	1	0.515
Same age				0.029	1	0.864				0.322	1	0.57				4.531	1	0.033
Ego age effect				0.003	1	0.957				0.206	1	0.65				1.762	1	0.184
Rate effect on sex on rate	0.66	0.22	-0.14	9.048	1	**0.003**	0.98	0.23	0.03	17.889	1	**<0.001**				0.406	1	0.524
Rate effect on matriline on rate				2.774	1	0.096	-0.1	0.03	0.08	12.276	1	**<0.001**				1.147	1	0.284
Rate effect on hierarchy on rate				0.716	1	0.397				0.53	1	0.466				0.741	1	0.389
Rate effect on age on rate				1.236	1	0.266				1.001	1	0.317	0.64	0.19	0.03	11.334	1	**<0.001**

Significant effects are represented in bold and the estimate, the standard error and the t ratio are also given for these effects.

For the homophily, the effect is considered as significant only if both main (i.e., sex, matriline, hierarchy, and age) and the ego effects are significant.

## Results

First of all, the goodness of fit analyses indicated that our model selection was reasonably accurate for all three groups, AK (MHD = 156.51; *P* = 0.054), BD (MHD = 126.65; *P* = 0.425), and NH (MHD = 77.17; *P* = 0.434).

When analyzing the structure of the network, all three groups showed a significant effect of triadic closure (AK: χ^2^ = 7.794; DF = 1; *p* = 0.029; BD: χ^2^ = 21.573; DF = 1; *P* < 0.001; NH: χ^2^ = 53.561; DF = 1; *P* < 0.001, Table 2), while there was a significant effect of degree popularity in only two groups (AK: χ^2^ = 3.918; DF = 1; *P* = 0.048; BD: χ^2^ = 4.228; DF = 1; *P* = 0.039; Table 2).

With respect to the structure, when analyzing the likelihood of homophilic bonds according to the different attributes, we could not find any general pattern across all three groups. Only the AK (χ^2^ = 8.615; DF = 1; *P* = 0.003) and the NH (χ^2^ = 21.719; DF = 1; *P* < 0.001) group members showed a significant preference of association to individuals of the same sex while preference of association with the same matriline was present only in the BD (χ^2^ = 41.833; DF = 1; *P* < 0.001) and NH (χ^2^ = 71.463; DF = 1; *P* < 0.001; Table 2) groups.

Finally, when looking at the network dynamics with relationships variation over time, results indicated a strong intergroup variation. In the AK groups, we found that females experience a greater and quicker relationships' variation than males do (χ^2^ = 9.048; DF = 1; *P* = 0.003) while for the BD group there was a significant effect of sex and matriline, suggesting that males' relationships are more prone to variation than females' (χ^2^ = 17.889; DF = 1; *P* < 0.001) and that high-ranking matrilines also experience a greater variation in their relationships stability (χ^2^ = 12.276; DF = 1; *P* < 0.001). Only in the NH group, we found that juveniles' relationships were more prone to variation than adults' (χ^2^ = 11.334; DF = 1; *P* < 0.001; Table 2).

## Discussion

In this study we tried to understand the dynamics of a social network through detailed analysis of the creation and destruction of relationships over time according to the following individual attributes: sex, matriline, hierarchy, and age. Main results indicate that individuals associate themselves with friends of their friends but many differences exist between the three groups. To our knowledge, this is the first study that uses a SAOM to analyze such dynamics on multiple and non-experimental groups. Indeed, another study (Ilany et al., 2015) already used such a model, but focused on only one group of hyenas and the effects of ecological variables. RSiena package was also used to understand social information transmission in experimental groups of drosophila (Pasquaretta et al., 2016). Our results show the importance of observing multiple groups when we want to assess the effect of different social variables on the temporal evolution of a network structure.

The analyses on triadic closure (which represents the likelihood of two individuals to be associated if they have a mutual third party associate) indicated that such effect was present in all three groups. According to some hypotheses, triadic closure might facilitate the evolution of cooperation (Banks and Carley, 1996; Davidsen et al., 2002; Righi and Takacs, 2014). For example, someone might be more likely to become friends with and potentially help a friend of a friend. This suggests that vervet monkeys' social system met the conditions for the emergence of triadic closure (Lusseau et al., 2006). In animals, only one study focused on how the triadic associations influence a social network structure (Ilany et al., 2013). However, what remains unknown with such theory is if triadic closure is the evolutionary consequence or the prerequisite of cooperation. More studies are needed to understand whether triadic closure is a by-product of social network or relatedness or is a social strategy leading to better cooperation between multiple partners. The degree popularity results, which represent the preferred association to highly central individuals, indicate that individuals try to bond with individuals that are central within a network, but this effect was found only in two groups. This pattern results in more centralized networks having great impact on information and disease transmission and several researches are done to understand whether and how natural selection might impact these social network properties (Pasquaretta et al., 2014; Duboscq et al., 2016; Romano et al., 2016). As multiple previous studies found a positive correlation between rank and/or matriline and centrality (Schino, 2001; Kanngiesser et al., 2011; Sueur et al., 2011a; Borgeaud et al., in preparation), our results partially support the generality of Seyfarth's model (1977). This model also suggests that grooming can be exchanged against tolerance among food resources or coalitionary support, which seems to exist in vervet monkeys (Borgeaud and Bshary, 2015). Central individuals are either high-ranking individuals, either close relatives or experienced individuals (Sueur et al., 2011b). In this way we can easily understand how preferred association to central individuals might be selected as a social strategy increasing fitness but still, we can observe that this effect is dependent on group composition. However, it should be noted that some studies fail to provide evidence for degree popularity, including in vervet monkeys (Henzi et al., 2013), as we do for one of the study groups.

We also tested if individuals associated preferably with individuals of similar attributes. After controlling for the differences in sex ratio (Female ratio: AK: 44; BD: 56; NH: 50%), our results surprisingly indicate that females form stronger bonds between themselves rather than with males only in the AK and NH groups. These results confirm that individuals of the philopatric sex, which normally remain in their natal group throughout their lives form strong and long-lasting bonds with each other (Cheney and Seyfarth, 1990; Silk et al., 2010). However, it remains challenging to explain the absence of significant results in the BD group. One explanation could rely on the presence of multiple adult males who, in contrary to the other groups were already present within the group at the beginning of the project in 2010 and remained within the group for a large part of the study. In this situation and at least on the time period of our study, females might have developed strong and long lasting relationships with these males. Similarly, our results suggest that members from the same matriline form stronger bonds than members of different matrilines, but only in the BD and NH groups. The positive results fit predictions by kin selection (Hamilton, 1964), while it remains unclear why such an effect should be absent in the AK group. In contrary to these two groups, the AK group is generally more tolerant and females of distant ranks regularly groom each other (Borgeaud and Bshary, 2015), which could reflect the results of this study. Tolerance between non kin was shown to be an advantage to decrease risk injuries, energy costs to maintain social relationships, or increase food research efficiency (Sueur et al., 2011a,b; Fushing et al., 2013; Pasquaretta et al., 2014). Preliminary results on genetics indicate that the average relatedness from the AK group members is 0.25 while both BD and NH are related at the level of 0.15 (Schnider et al., unpublished data). These results support previous results indicating that kin form stronger bonds than non kin individuals (Silk et al., 2010). We did not find any effect of hierarchy on bonds' strength. This suggests that individuals of close ranks either do not have stronger bonds than individuals of distant ranks or they have stronger bonds but this effect is undone by the more important effects of sex and matriline. As our analyses include both males and females, another explanation could be that high-ranking males may bond as much with high-ranking than with low-ranking females, canceling a potential rank effect. Finally, our lack of results about association between individuals of similar age is rather surprising as this difference cannot really be explained by a difference in age ratio (Adult age ratio: AK: 43; BD: 31; NH: 42%). Previous studies suggested the importance of bonds with individuals of similar age. For example juveniles' play-fights allow the development of the social techniques necessary to acquire a central position in a society (Shimada and Sueur, 2014). On the other hand it might simply reflect that, despite the age difference, bonds between a mother and her offspring are the strongest of all associations, which has also been found in baboons (Silk et al., 2010). Another explanation could rely on the fact that our juvenile age category included 1–4 years olds and it is likely that they form stronger bonds within rather than across generations.

Finally, when testing how quickly relationships are modified according to the individual attributes, we found no patterns that were consistent among our three groups. In both AK and BD groups, females' relationships are more prone to variation than the males' and in BD the relationships of individuals belonging to high-ranking matrilines were also less stable. This supports the Seyfarth's model (1977) which implies a potential instability of higher ranking individuals' relationships due to social competition. The BD group was the only one where the high-ranking matrilines had a significant influence on how quickly relationships were modified. Similarly, previous studies found differences between populations in their relationships management (Silk et al., 1999; Henzi et al., 2013). Finally, in the NH group, our results suggest that adults' relationships are more stable than those of juveniles. These results support previous studies in baboons (Silk et al., 2006a,b, 2010, 2012), which indicate stable relationships within adult females. Female juveniles in vervet monkeys form strong and rather stable relationships with adult females while male juveniles' relationships are more prone to variation (Fairbanks, 2002; juvenile vervet monkeys). However, the fact that such results are significant only in one group is rather puzzling but could be due to group differences in relationships management and group composition (Cronin et al., 2014a,b).

We based our evaluation of effect size entirely on the distinction “significant effect” vs. “non-significant effect” and the size of the estimate. In the future, it would be interesting to test multiple groups simultaneously following the “multilevel” SAOM method that has been recently developed (Snijders et al., 2013). To our knowledge this is the first time that a study focuses on the social network dynamics of three different groups of monkeys. Interestingly, our results indicate substantial intergroup variation. This variation might be due to (1) real intergroup difference, (2) problem in methodology, (3) non powerful statistical analyses. However, we made considerable efforts to apply the same scoring methods on the three groups. Despite this effort, various *p*-values were either non-significant or so very small (< 0.001) and seems to indicate that groups differed indeed with respect to various variables. However, we currently cannot test how much intergroup variation could be due to differences in genetic relatedness. On the other hand, a purely ecological explanation seems unlikely as all three groups live in overlapping home ranges. In part, the differences could also be due to different individual strategies and/or personalities, which could have various impacts on the network variation depending of their position within this network (Cronin et al., 2014a,b). Such a cause of variation would indeed be interesting. In any case, our results suggest that studies on multiple groups are necessary to build up any hypothesis concerning network features and dynamics within a species.

Most primates live in closely related and bonded social groups in which individuals have to deal with many social challenges and opportunities (Humphrey, 1976; Harcourt, 1988). Famously, Humphrey (1976) proposed that large brains evolved in primates primarily to cope with the social environment as it is less predictable than the physical environment. This idea has been developed further in the Machiavellian intelligence and social brain hypotheses (Byrne and Whiten, 1988; Dunbar, 1992; Whiten and Byrne, 1997). Therefore, the complexity of a species' social network may be a good indicator for the cognitive demands that individuals face and be reflected in the complexity of the species' brain. To be able to test this hypothesis, we first need to establish methods on how to measure different aspects of network complexity (Lehmann and Dunbar, 2009). The methods we used rely on quantifying the dynamics of relationships patterns according to individual attributes while considering changes in group composition. These analyses could be applied to a variety of species. Ultimately such measures should allow a comparison between species to assess how network dynamics is correlated with brain complexity. In this context, the observed variation among group network structures may turn out to be an indicator of great social flexibility that demands a social brain.

## Ethics statement

The study was approved by the relevant authority, Ezemvelo KZN Wildlife and by the University of Cape Town, South Africa.

## Author contributions

Data collection: CB and Ev; writing: first version by CB, then all authors contributed; statistical analyses: SS and CS; funding for data collection by SNF Sinergia grant to RB.

### Conflict of interest statement

The authors declare that the research was conducted in the absence of any commercial or financial relationships that could be construed as a potential conflict of interest.

## Supplementary material

Supplementary Figure 1 **Goodness of fit plots for the degree distribution, the geodesic distribution, and the triad census for each group**.
