# Supplementary figures and images for: Intergroup Variation of Social Relationships in Wild Vervet Monkeys: A Dynamic Network Approach
Source: Front Psychol. 2016 Jun 21;7:915. doi: 10.3389/fpsyg.2016.00915 (PMC4914564; doi:10.3389/fpsyg.2016.00915)

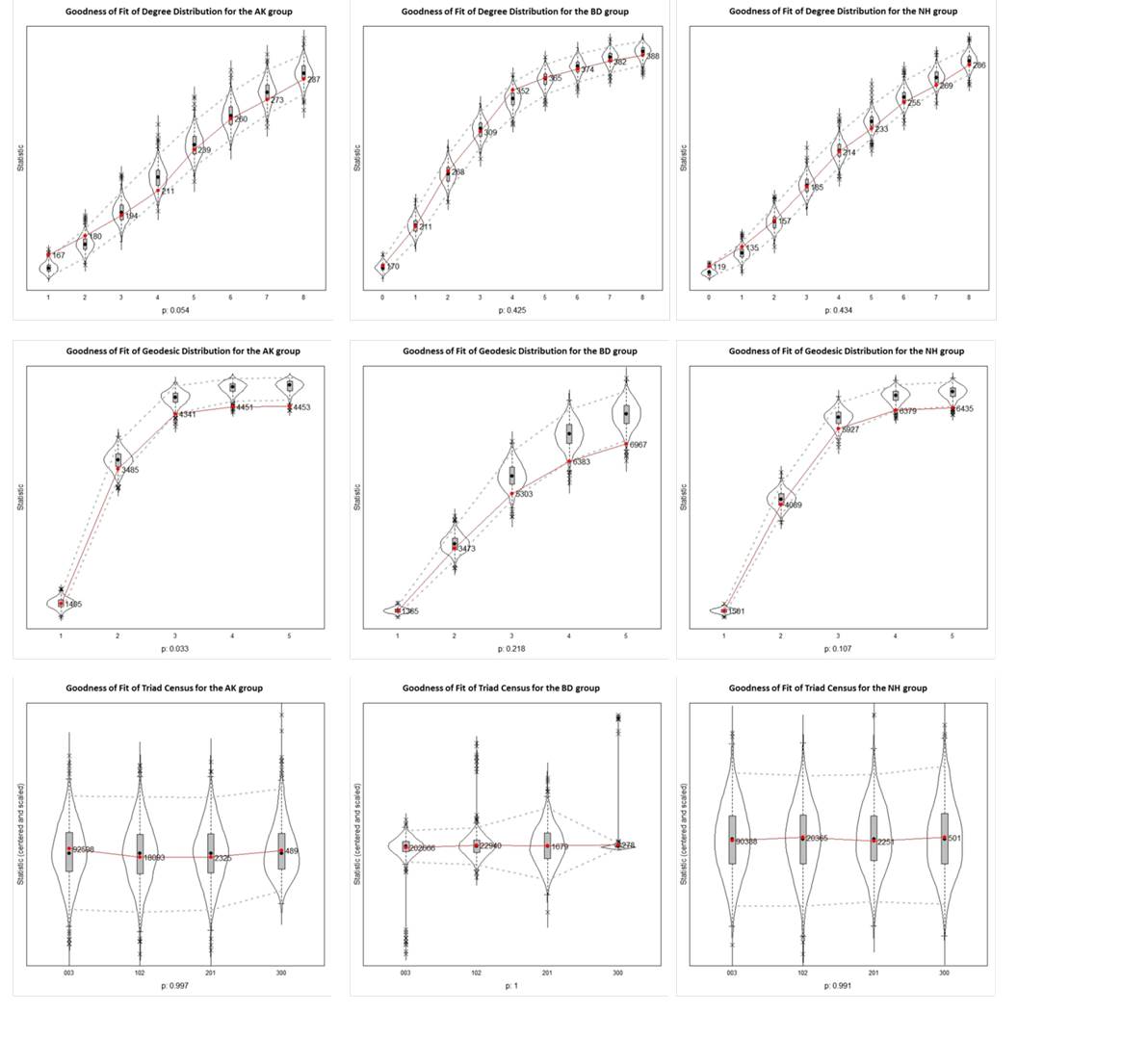

Supplement: Supplementary Figure 1 — Goodness of fit plots for the degree distribution, the geodesic distribution, and the triad census for each group. [file Image2.JPEG]
