# Supplementary material for: Intergroup Variation of Social Relationships in Wild Vervet Monkeys: A Dynamic Network Approach
Source: Front Psychol. 2016 Jun 21;7:915. doi: 10.3389/fpsyg.2016.00915 (PMC4914564; doi:10.3389/fpsyg.2016.00915)
Supplement: Supplementary file 2 [file DataSheet1.DOCX]

library(RSiena)

library( network )

library( sna )

library(xtable)

siena07ToConvergence <- function(alg, dat, eff, ans0=NULL, ...){

numr <- 0

ans <- siena07(alg, data=dat, effects=eff, prevAns=ans0, ...)

repeat {

numr <- numr+1

tm <- ans$tconv.max

cat(numr, tm,"\n")

if (tm < 0.25) {break}

if (tm > 8) {break}

if (numr > 100) {break}

ans <- siena07(alg, data=dat, effects=eff, prevAns=ans, ...)

}

if (tm > 0.25)

{

cat("Warning: convergence inadequate.\n")

}

ans

}

#Auxiliary statistics

DegreeDistribution<-function (i, obsData, sims, period, groupName, varName, levls = 0:8,

cumulative = TRUE)

{

x <- sparseMatrixExtraction(i, obsData, sims, period, groupName,

varName)

a <- apply(x, 1, sum)

if (cumulative) {

oddi <- sapply(levls, function(i) {

sum(a <= i)

})

}

else {

oddi <- sapply(levls, function(i) {

sum(a == i)

})

}

names(oddi) <- as.character(levls)

oddi

}

GeodesicDistribution <- function (i, data, sims, period, groupName,

varName, levls=c(1:5,Inf), cumulative=TRUE, ...) {

x <- networkExtraction(i, data, sims, period, groupName, varName)

require(sna)

a <- sna::geodist(x)$gdist

if (cumulative)

{

gdi <- sapply(levls, function(i){ sum(a<=i) })

}

else

{

gdi <- sapply(levls, function(i){ sum(a==i) })

}

names(gdi) <- as.character(levls)

gdi

}

TriadCensus <- function(i, data, sims, wave, groupName, varName, levls=1:16){

unloadNamespace("igraph") # to avoid package clashes

require(sna)

require(network)

x <- networkExtraction(i, data, sims, wave, groupName, varName)

tc <- sna::triad.census(x)[1,levls]

tc

}

NHfriend.data.w1 <- as.matrix(read.table("NH1.csv"))

NHfriend.data.w2 <- as.matrix(read.table("NH2.csv"))

NHfriend.data.w3 <- as.matrix(read.table("NH3.csv"))

NHfriend.data.w4 <- as.matrix(read.table("NH4.csv"))

NHfriend.data.w5 <- as.matrix(read.table("NH5.csv"))

NHfriend.data.w6 <- as.matrix(read.table("NH6.csv"))

NHfriend.data.w7 <- as.matrix(read.table("NH7.csv"))

NHfriend.data.w8 <- as.matrix(read.table("NH8.csv"))

NHGender<- as.matrix(read.table("NHGenre.csv",na.strings="NA"))

NHAge<- as.matrix(read.table("NHAge.csv",na.strings="NA"))

NHHierarchy<- as.matrix(read.table("NHHierarchy.csv",na.strings="NA"))

NHMatrilines<- as.matrix(read.table("NHmatriligne.csv",na.strings="NA"))

NHsex.M <- coCovar(NHGender[,1])

NHMatriline <- coCovar(NHMatrilines[,1])

NHAge <- varCovar(NHAge)

NHHierarchy <- varCovar(NHHierarchy)

NHfriendship <- sienaDependent(array( c( NHfriend.data.w1, NHfriend.data.w2, NHfriend.data.w3,NHfriend.data.w4,NHfriend.data.w5,NHfriend.data.w6,NHfriend.data.w7,NHfriend.data.w8),dim = c( 47,47, 8 ) ) )

NHmydata <- sienaDataCreate( NHfriendship,NHsex.M,NHAge,NHHierarchy,NHMatriline)

NHmyeff <- getEffects(NHmydata)

NHmyeff <- includeEffects(NHmyeff,transTriads)

NHmyalgorithm <- sienaAlgorithmCreate( projname = 'NH + transTrip')

ans1 <- siena07ToConvergence( NHmyalgorithm,NHmydata,NHmyeff,returnDeps=TRUE)

ans1

NHTrasTripEffect<-Multipar.RSiena(ans1,2)

NHTrasTripEffect

NHmyeff <- includeEffects(NHmyeff,inPop,include=FALSE)

NHmyalgorithm <- sienaAlgorithmCreate( projname = 'NH + transTrip'+inPop)

ans2 <- siena07ToConvergence( NHmyalgorithm,NHmydata,NHmyeff,returnDeps=TRUE)

ans2

NHInPopEffect<-Multipar.RSiena(ans2,3)

NHInPopEffect

NHmyeff<- includeEffects(NHmyeff,sameX,egoXaltX, interaction1 = "NHAge")

NHmyalgorithm <- sienaAlgorithmCreate( projname = 'NH + inPop+transTrip+SameAge')

ans3 <- siena07ToConvergence( NHmyalgorithm,NHmydata,NHmyeff,returnDeps=TRUE)

ans3

NHSameAgeEffect<-Multipar.RSiena(ans3,4)

NHEgoAltAgeEffect<-Multipar.RSiena(ans3,5)

NHSameAgeEffect

NHEgoAltAgeEffect

NHmyeff<- includeEffects(NHmyeff,sameX,egoXaltX, interaction1 = "NHAge",include=FALSE)

NHmyeff<- includeEffects(NHmyeff,sameX,egoXaltX, interaction1 = "NHsex.M")

NHmyalgorithm <- sienaAlgorithmCreate( projname = 'NH inPop+transTrip+SameSex')

ans4 <- siena07ToConvergence( NHmyalgorithm,NHmydata,NHmyeff,returnDeps=TRUE)

ans4

NHSameSexEffect<-Multipar.RSiena(ans4,4)

NHEgoAltSexEffect<-Multipar.RSiena(ans4,5)

NHSameSexEffect

NHEgoAltSexEffect

NHmyeff<- includeEffects(NHmyeff,sameX,egoXaltX, interaction1 = "NHMatriline")

NHmyalgorithm <- sienaAlgorithmCreate( projname = 'NH + inPop+transTrip+SameSex+SameMatriline')

ans5 <- siena07ToConvergence( NHmyalgorithm,NHmydata,NHmyeff,returnDeps=TRUE)

ans5

NHSameMatrilineEffect<-Multipar.RSiena(ans5,6)

NHEgoAltMatrilineEffect<-Multipar.RSiena(ans5,7)

NHSameMatrilineEffect

NHEgoAltMatrilineEffect

NHmyeff<- includeEffects(NHmyeff,simX,egoXaltX, interaction1 = "NHHierarchy")

NHmyalgorithm <- sienaAlgorithmCreate( projname = 'NH + inPop+transTrip+SameSex+SameHierarchy')

ans6 <- siena07ToConvergence( NHmyalgorithm,NHmydata,NHmyeff,returnDeps=TRUE)

ans6

NHSameHierarchyEffect<-Multipar.RSiena(ans6,8)

NHEgoAltHierarchyEffect<-Multipar.RSiena(ans6,9)

NHSameHierarchyEffect

NHEgoAltHierarchyEffect

NHmyeff<- includeEffects(NHmyeff,simX,egoXaltX, interaction1 = "NHHierarchy",include=FALSE)

NHmyeff<- includeEffects(NHmyeff, RateX, type="rate",interaction1="NHsex.M")

NHmyalgorithm <- sienaAlgorithmCreate( projname = 'NH + inPop+transTrip+SameSex+RateSex')

ans7 <- siena07ToConvergence( NHmyalgorithm,NHmydata,NHmyeff,returnDeps=TRUE)

ans7

NHSexRateEffect<-Multipar.RSiena(ans7,1)

NHSexRateEffect

NHmyeff<- includeEffects(NHmyeff, RateX, type="rate",interaction1="NHsex.M",include=FALSE)

NHmyeff<- includeEffects(NHmyeff, RateX, type="rate",interaction1="NHMatriline")

NHmyalgorithm <- sienaAlgorithmCreate( projname = 'NH + inPop+transTrip+SameSex+RateSex+RateMatriline')

ans8 <- siena07ToConvergence( NHmyalgorithm,NHmydata,NHmyeff,returnDeps=TRUE)

ans8

NHMatrilineRateEffect<-Multipar.RSiena(ans8,1)

NHMatrilineRateEffect

NHmyeff<- includeEffects(NHmyeff, RateX, type="rate",interaction1="NHMatriline",include=FALSE)

NHmyeff<- includeEffects(NHmyeff, RateX, type="rate",interaction1="NHAge")

NHmyalgorithm <- sienaAlgorithmCreate( projname = 'NH transTrip+inPop+SameSex+SameMatriline+RateAge')

ans9 <- siena07ToConvergence( NHmyalgorithm,NHmydata,NHmyeff,returnDeps=TRUE)

ans9

NHAgeRateEffect<-Multipar.RSiena(ans9,1)

NHAgeRateEffect

NHmyeff<- includeEffects(NHmyeff, RateX, type="rate",interaction1="NHHierarchy")

NHmyalgorithm <- sienaAlgorithmCreate( projname = 'NH transTrip+inPop+SameSex+SameMatriline+RateAge+RateHierarchy')

ans10 <- siena07ToConvergence( NHmyalgorithm,NHmydata,NHmyeff,returnDeps=TRUE)

ans10

NHHierarchyRateEffect<-Multipar.RSiena(ans10,2)

NHHierarchyRateEffect

NHmyeff<- includeEffects(NHmyeff, RateX, type="rate",interaction1="NHHierarchy",include=FALSE)

NHmyalgorithm <- sienaAlgorithmCreate( projname = 'NH transTrip+inPop+SameSex+SameMatriline+RateAge')

NHans11<- siena07ToConvergence( NHmyalgorithm,NHmydata,NHmyeff,returnDeps=TRUE)

NHans11

NHTrasTripEffect<-Multipar.RSiena(NHans11,3)

NHInPopEffect<-Multipar.RSiena(NHans11,4)

NHSameSexEffect<-Multipar.RSiena(NHans11,5)

NHEgoAltSexEffect<-Multipar.RSiena(NHans11,6)

NHSameMatrilineEffect<-Multipar.RSiena(NHans11,7)

NHEgoAltMatrilineEffect<-Multipar.RSiena(NHans11,8)

NHAgeRateEffect<-Multipar.RSiena(NHans11,1)

NHTrasTripEffect

NHInPopEffect

NHSameSexEffect

NHEgoAltSexEffect

NHSameMatrilineEffect

NHEgoAltMatrilineEffect

NHAgeRateEffect

DgofNH.id <- sienaGOF(NHans11, verbose=TRUE, varName="NHfriendship", DegreeDistribution)

GgofNH.id <- sienaGOF(NHans11, verbose=TRUE, varName="NHfriendship", GeodesicDistribution)

TgofNH.id <- sienaGOF(NHans11, verbose=TRUE, varName="NHfriendship", TriadCensus)

DgofNH.id

GgofNH.id

TgofNH.id

plot(DgofNH.id, center=FALSE, scale=FALSE, violin=TRUE, key=NULL,perc=.05)

plot(GgofNH.id, center=FALSE, scale=FALSE, violin=TRUE, key=NULL,perc=.05)

plot(TgofNH.id, center=TRUE, scale=TRUE)

#Sauvegarder en format html

xtable(NHans11, type='html',file='NH.html')
